# Demographic Differences in the Surgical Management of Tibial Shaft Fractures: A Retrospective Study

**DOI:** 10.7759/cureus.78917

**Published:** 2025-02-12

**Authors:** Tyler Beaudoin, Mustafa Hashimi, Avery Allen, Michael Hawks, Atif Ahmed, Benjamin D Sookhoo, Kassem Ghayyad

**Affiliations:** 1 Orthopaedic Surgery, Rothman Orthopaedics Florida at AdventHealth, Orlando, USA

**Keywords:** compound tibia diaphyseal fractures, fractures of the tibia, interlocking nail tibia, intramedullary nailing of the tibia, open tibia reduction, tibia diaphysis, tibia nailing, tibia non-union, tibia shaft, tibia shaft fracture

## Abstract

Background

Tibial shaft fractures (TSFs) are the most common long bone fractures in the United States and are associated with significant morbidity and the potential need for revision surgeries, with many patients requiring reoperation. This can have significant physical, mental, and financial impacts on patients. A major complication faced by patients with TSF is nonunion (TSFN). The mainstay of surgical management of TSF is Intramedullary Nail (IMN), with some patients also being treated with Open Reduction Internal Fixation (ORIF). With the demographic makeup of the United States undergoing rapid change, a better understanding of patient characteristics of patients with TSF is useful to optimize patient care. This study aims to enhance our comprehension of the frequency and demographic variables associated with tibia fracture surgery and subsequent nonunion.

Methods

A retrospective study was conducted in August 2023, utilizing the TriNetX “Global Collaborative Network” database to form patient study cohorts. Data extracted included patient age, sex, ethnicity, race, smoking status, surgical management, and nonunion. Data was also extracted on specific surgical management utilized, comprising either IMN or ORIF.

Results

A total of 6,389 cases of TSFs were analyzed, with 65% (4,153) of patients undergoing ORIF compared to IMN (35%, or 2,236). The overall incidence of patients with TSF ORIF and IMN was highest among males and White patients. The incidence of patients with TSF ORIF was highest in the age groups of 40-64 and 64-90 years, while TSF IMN was highest in the age groups of 18-39 and 40-64 years. The overall rate of tibia nonunion among patients with a TSF ORIF was 4.6%, vs. 2.6% in patients who underwent IMN.

Conclusion

TSFs treated with IMN were found to have lower rates of nonunion compared to ORIF. IMN of TSF was more common in younger patients, while ORIF was more common in the older age groups. ORIF and IMN had similar rates of male-to-female utilization, indicating that both genders are being treated similarly with regard to the operative method of choice. White patients and those who were not Hispanic or Latino had much higher rates of both IMN and ORIF compared to all other racial groups, despite the increased complexity of fracture/injury characteristics and higher complication rates in minority patients.

## Introduction

Tibial shaft fractures (TSFs) are the most common long bone fracture, with rates of fracture upwards of 20 per 100,000 people in the United States, compared to other long bone fractures such as femur shaft fractures, which have a rate closer to 10 per 100,000 [1-4]. Additionally, TSF accounts for 2% of all fractures in adult patients [5]. TSFs pose a significant burden on the healthcare system, as they result in over 800,000 office visits and 70,000 hospitalizations in the United States each year, with large socioeconomic ramifications for patients and the general healthcare system [6]. The mechanism of injury for TSF is usually high-energy motor vehicle collisions (MVCs) in younger adults, while lower-energy injuries, like mechanical ground-level falls, cause this in older adults [7,8]. The age distribution of TSF also follows a bimodal distribution, peaking around age 20 and also around age 50 [7]. TSF is associated with significant morbidity, such as acute and chronic lower extremity pain, compartment syndrome, and nonunion. The morbidity of TSF can place significant financial burdens on patients, especially since such high rates of fractures are seen in working-age adults, which can impact their ability to work and support themselves and their families [9,10]. The mainstays of surgical management of TSF are either Intramedullary Nail (IMN) or Open Reduction Internal Fixation (ORIF) [11]. A major complication faced by patients with TSF is tibial shaft fracture nonunion (TSFN), with about 5%-20% incidence even after surgical management [12-16]. This leads to significant physical, mental, and financial hardship for patients. Patients with TSFN have double the healthcare costs compared to patients with TSF that unite uneventfully, costing over $14,000 more [17]. With the demographic makeup of the United States undergoing rapid change, a better understanding of the characteristics of patients who suffer from TSF and TSFN is useful in optimizing patient care.

This study aims to leverage a global network database, called TriNetX, to enhance our comprehension of the frequency and demographic variables associated with tibia fracture surgery and subsequent nonunion.

## Materials and methods

A retrospective study was conducted in the United States in August 2023, utilizing the TriNetX “Global Collaborative Network” database on patients who had surgical intervention of their TSF from 2017 to 2022 within the United States. The use of the TriNetX database does not involve patient-identifiable information and is, subsequently, Institutional Review Board exempt.

Patient cohort identification

Patient cohorts were defined using the International Classification for Disease, 10th Edition (ICD-10) diagnosis codes. Patients diagnosed with TSFs (S82.201A, S82.202A) and TSFN (S82.201K, S82.202K) between January 1, 2017, and December 31, 2022, were included. A total of 58,019 patients with TSFs and 2,100 patients with tibia nonunion were identified.

Surgical procedure identification

Patients with TSF ORIF (Current Procedural Terminology (CPT) code: 27758) and TSF IMN (CPT code: 27784) were searched. Next, patients were searched chronologically for TSFN (ICD-10 code: S82.201K, S82.202K) to further stratify the surgical cohorts, with nonunion diagnosed between January 1, 2017, and December 31, 2022, included.

Demographical features

Patients’ information was extracted, including age, sex, ethnicity, race, and smoking status. Age was divided into four quartiles: 0-17, 18-39, 40-64, and 65-90. Ethnicity was divided into Hispanic or Latino, Not Hispanic or Latino, and unknown. Race was divided into White patients, Black patients, Asian patients, Native American or Pacific Islander patients, American Indian patients or Alaska Native patients, and unknown patients.

Global collaborative network

The Global Collaborative Network through TriNetX is a web-based database tool, allowing for population cohort research, feasibility queries, and collaboration with medical researchers worldwide. The database contains an extensive network of over 400 million de-identified patient data that can be accessed on demand without prior Institutional Review Board (IRB) approval. The database allows access to patient demographics, diagnoses, procedures, labs, and medications. Data is obtained through collaboration between over 200 community- and academic-based healthcare organizations and industry partners worldwide. The data that was pulled from this database for the study includes unidentified patient data from electronic medical records used at various health systems within the United States. 

## Results

Tibia fracture ORIF

A total of 4,153 TSFs ORIF from 2017 to 2022 were analyzed from the dataset. The overall incidence of patients with TSFs ORIF was highest in the age groups of 40-64 (35.5%) and 64-90 (28.9%) years, and lowest in the age groups of 0-17 (9.1%) and 18-39 (26.5%). TSFs ORIF were more common in males (2,167 patients) than females (1,498 patients), with a ratio of M/F = 1.44/1. Among patient ethnicities, TSFs ORIF incidence was higher among those not Hispanic or Latino (2,825) than those who were Hispanic or Latino (841), with a ratio of 3.35/1. Fractures were more common among White patients (2,655) than the following highest incidence groups: Black patients (504) or Asian patients (78) (Table 1).

**Table 1:** Demographics of patients underwent tibia fracture ORIF Tibia ORIF (Current Procedural Terminology (CPT) code: 27758) ORIF: Open reduction internal fixation

Year	2017	2018	2019	2020	2021	2022	2017-2022
Total patients	534	665	659	689	820	786	4153
Age							
Mean age ± SD	52 ± 21.6	50.8 ± 21	51.4 ± 22	49.5 ± 21.2	47.2 ± 22	46.3 ± 21.5	49.2 ± 21.7
0-17	15	29	50	55	108	121	378
18-39	153	183	166	183	221	195	1101
40-64	173	259	244	258	264	276	1474
65-90	193	194	199	193	227	194	1200
Sex							
Female	210	221	238	238	315	276	1498
Male	255	357	346	375	424	410	2167
Unknown	69	87	75	76	81	100	488
Ethnicity							
Not Hispanic or Latino	363	413	463	477	584	525	2825
Unknown	117	150	118	145	148	163	841
Hispanic or Latino	54	102	78	67	88	98	487
Race							
White patients	360	430	437	439	506	483	2655
Unknown patients	92	132	114	132	133	148	751
Black patients	66	70	67	84	119	98	504
Asian patients	10	10	12	10	22	14	78
Native Hawaiian or other Pacific Islander patients	10	10	10	10	10	0	50
American Indian or Alaska Native patients	0	10	10	0	10	0	30
Smoking Hx (z87.891)							
Yes	105	121	136	128	146	146	782
No	429	544	523	561	674	640	3371

Tibia fracture nonunion following ORIF

The overall rate of tibia nonunion among patients with a TSF ORIF was 4.6% (191/4,153), between 2017 and 2022 (Table 2).

**Table 2:** Nonunion rate following tibia fracture ORIF ORIF: Open reduction internal fixation

Year	2017	2018	2019	2020	2021	2022	2017-2022
Total patients	31	38	34	36	25	27	191

Tibia fracture IMN

We analyzed the frequency data of 2,236 TSFs IMN from 2017 to 2022 (Table 3). The overall incidence of patients with TSFs IMN was highest in the age groups of 18-39 and 40-64 years, and lowest in the age groups of 0-17 and 65-90. TSFs IMN were more common in males (1,227 patients) than females (745 patients), with a ratio of M/F = 1.64/1. Among patient ethnicities, TSF IMN incidence was higher among those not Hispanic or Latino (1,539) than those who were Hispanic or Latino (490), with a ratio of 3.14/1. TSFs IMN were more common among White patients (1,357) than the following highest incidence groups: Black patients (345) or Asian patients (60).

**Table 3:** Demographics of patients who underwent tibia fracture IMN Tibia IMN (Current Procedural Terminology (CPT) code: 27784) IMN: Intramedullary nail

Year	2017	2018	2019	2020	2021	2022	2017-2022
Total patients	357	326	393	387	419	354	2236
Age							
Mean age ± SD	48.6 ± 17.6	48 ± 17.5	47.2 ± 17.3	45.3 ± 17.8	45.5 ± 17.6	42.4 ± 17.4	46.1 ± 17.6
0-17	2	3	0	5	14	11	35
18-39	122	119	152	167	162	161	883
40-64	163	136	164	150	179	138	930
65-90	70	68	77	65	64	44	388
Sex							
Female	122	98	130	140	129	126	745
Male	184	184	222	203	242	192	1227
Unknown	51	44	41	44	48	36	264
Ethnicity							
Not Hispanic or Latino	255	216	270	256	295	247	1539
Unknown	80	76	77	96	90	71	490
Hispanic or Latino	22	34	46	35	34	36	207
Race							
White patients	211	189	243	236	257	221	1357
Unknown patients	68	69	59	76	61	59	392
Black patients	62	47	60	58	74	44	345
Asian patients	10	10	10	10	10	10	60
Native Hawaiian or other Pacific Islander patients	0	10	10	0	0	10	30
American Indian or Alaska Native patients	0	10	10	0	0	0	20
Smoking Hx (z87.891)							
Yes	66	63	64	57	62	38	350
No	291	263	329	330	357	316	1886

Tibia fracture nonunion following IMN

The overall rate of tibia nonunion among patients with TSFs IMN was 2.6% (59/2,236), between 2017 and 2022 (Table 4).

**Table 4:** Nonunion rate following tibia fracture IMN Tibia IMN nonuion (S82.201K, S82.202K) IMN: Intramedullary nail

Year	2017	2018	2019	2020	2021	2022	2017-2022
Total patients	13	10	17	10>	10>	10>	59

## Discussion

To improve patient outcomes for those suffering from TSF and TSFN undergoing IMN or ORIF, it is important for orthopedic surgeons to have a better understanding of demographic factors that impact the patient's risk of nonunion. This understanding can help in counseling patients, planning operative interventions, and identifying those at risk of complications. This study identified multiple findings regarding the demographics of patients who undergo either IMN or ORIF, and subsequent nonunion.

The vast majority of patients with TSF are treated surgically, while only 12% of patients are treated nonoperatively, with the treatment of choice for TSF being IMN [18]. Overall, in patients with TSF, a study by Wennergren et al. found that 71% are treated with primary IMN, while ORIF was much less commonly used, which is a finding also found in various other studies [18-20]. The high rates of primary surgical management of TSF may explain the relatively low rates of TSFN [21]. Several studies have demonstrated that surgical management of TSF with IMN or ORIF is associated with lower rates of nonunion and malalignment compared to nonoperative management. This suggests low nonunion rates, as the majority of patients are being treated surgically [8,21-23].

This study found that most patients in the included cohort were treated with ORIF as opposed to IMN for TSF, with 65% of patients undergoing ORIF. This finding differs from the findings of other studies that identified IMN as the mainstay for surgical management of TSF [11,19,20]. This difference in findings may be due to differences in surgeon preference in the locations sampled in this study, as, although IMN may be the most commonly used surgical management, the best overall management is still a subject of debate [24]. The risk of fracture malalignment has been found to be higher with IMN compared to ORIF, with rates up to 58% with IMN, as well as higher rates of anterior knee pain, which may lead more surgeons to opt for ORIF. This could be a potential explanation for the findings in the study [24-27]. However, IMN has generally been found to have higher union rates, lower infection rates, and earlier weight-bearing than ORIF, which generally makes it the preferred operative method [11,28-30].

The study demonstrated that TSF managed with IMN had lower rates of nonunion compared to ORIF, with IMN having a nonunion rate of 2.6%, compared to ORIF of 4.5%. This finding aligns with the findings of other studies that also found IMN to have lower nonunion rates compared to traditional ORIF [11,19,28-30]. One study by Shen and Tejwani found that ORIF of TSF had 2.52 greater odds of complication and 2.5 times the rate of nonunion compared to IMN [31]. However, some studies suggest that using Minimally Invasive Percutaneous Plate Osteosynthesis (MIPO) has the potential to bring ORIF in line with the lower complication rate of IMN, and possibly even surpass the union rate of IMN, and presents an opportunity for further research [24,25,32].

This study showed that IMN of TSF was more common in younger patients in age subgroups 18-39 and 40-64. ORIF was more common in the older age groups of 40-64 and 64-90. Upfill-Brown et al. found that the majority of patients with TSF treated with IMN were below the age of 50, compared to ORIF [11]. This finding may be due to TSF IMN having higher rates of reoperation compared to ORIF, with rates of reoperation after IMN upwards of 40% being reported, and a desire to use ORIF to reduce reoperation rates in older patients [12,33-35]. Additionally, a study by Bhandari et al. found that open fractures were a main predictor of the need for subsequent reoperation of TSF, and older patients have been found to have high rates of open fractures [33,36]. This trend may push surgeons to prefer ORIF in older patients to reduce reoperation rates and overall intraoperative time in patients who are older and may suffer from more comorbidities that increase surgical risk. Another possible explanation is that the rate of TSFN occurs more commonly in younger patients. Mills et al. found that in lower extremity long bone fractures, the rate of nonunion was highest among patients aged 30-44, and was significantly higher than rates in elderly patients, who are traditionally thought to suffer from higher rates of nonunion [37]. With TSF IMN having been found to have overall lower rates of nonunion, utilizing this surgical method in younger patients predisposed to nonunion might underlie the higher rates of IMN in the study groups of younger patients.

With regards to patient gender, males had higher rates of both IMN and ORIF of TSF when compared to female patients, which is likely attributed to higher overall TSF among male patients. The finding of higher rates of TSF in males compared to females follows the results of other studies that also displayed similar findings [2,5,7,38-40]. This trend was largely attributed to the higher rates in younger male patients with TSF secondary to higher energy injuries, such as MVCs or sports, while females suffered from TSF at older ages, at lower rates, mainly due to low-impact falls [2,7]. This trend in the mechanism of injury may also explain the higher rates of surgical management in male patients, as their injuries are more complex and have a higher rate of nonunion. Audigé et al. found that TSF associated with complex fracture patterns or open fractures were more likely to occur in the setting of high-energy trauma, such as traffic accidents, and these patients were more commonly males [16]. Additionally, a study by Mundi et al. found that TSF patients with open or complex fracture patterns had over a twofold increased risk of nonunion [3]. A separate study by Karladani et al. found that a patient with a TSF secondary to high-energy trauma had 2.9 times the risk of fracture nonunion, as well as soft tissue compromise. Exposure to high-energy trauma was a predictor of TSF complications [14]. With males suffering from high rates of TSF due to high-energy trauma with injury patterns that are at higher risk of nonunion, they are more likely to undergo surgical management. However, both ORIF and IMN have similar rates of male-to-female utilization, with a ratio of male to female of 1.64/1 with IMN and 1.44/1 with ORIF, indicating both genders are being treated similarly with regards to the operative method of choice.

Despite advances in the diagnosis and treatment of orthopedic medical conditions in the United States, significant racial disparities still exist between different races and ethnic groups [41,42]. This study found that White patients and those who were not Hispanic or Latino had much higher rates of both IMN and ORIF compared to all other racial groups. Racial disparities among outcomes and care for orthopedic injuries are well documented, with Black and minority patients receiving lower rates of surgical management and higher complication rates than White patients among many injury patterns [43-46]. Specifically regarding TSF disparities, a study by DeBaun et al. looked at disparities among Medicaid patients with TSF and found that Black patients had higher rates of more severe and open TSF, were less likely to fill postoperative prescriptions, and had lower rates of nonunion repair or management of complications compared to White patients [47]. The finding of lower reoperation rates and medical care of complications from initial TSF, despite DeBaun et al.'s study showing similar rates of complications among the two groups, as well as lower rates of prescriptions being filled, makes it possible that a disparity exists in access to care. This finding might indicate that Black patients overall may not have good access to care and may not be undergoing surgical management of TSF due to cost or other socioeconomic concerns, with significant disparities in access still present in the United States based on racial group [41]. A study by Landau et al. found that Black patients were 23% less likely to undergo surgical management, and those with Medicaid were 20% less likely to undergo surgical management of TSF than their counterparts [48]. DeBaun et al.'s study also found that Black patients with TSF were more likely to be younger, male, and suffer from more severe TSF than White patients [47]. With high-energy trauma and open fractures more likely to necessitate surgical management of TSF, having lower overall rates of IMN or ORIF among minority patients in this study may further indicate a disparity is present with regard to TSF surgical management [3,14]. Another possible explanation for the finding is that minority patients, and those with Medicaid or without insurance, may also be referred to other lower-quality practices for care and may not have their final management recorded within the study data [49]. 

The risk of fracture nonunion with tobacco use has been well documented, with tobacco reducing the critical blood flow needed to allow proper fracture healing [50,51]. Carbon monoxide and nicotine are postulated to be the main drivers of nonunion [50,51]. This study found that patients who smoked comprised a similar proportion of the study cohort - 15% of IMN and 18% of ORIF. This indicates that there was not a major difference in the selection of ORIF vs. IMN among smokers. This follows the findings of other studies that did not find a matched difference in outcomes among patients who smoked with either IMN or ORIF of their TSF [11,52].

The TriNetX database used in this study gives access to a broad variety of data from numerous healthcare organizations and hospital systems across the United States. This dataset was utilized to create a heterogeneous group of data that allowed data analysis that would not be available to a single healthcare organization in one geographic location. However, this study does have some limitations that are noted. The data does not include the mechanism of injury, fracture pattern, functional status, or outcome. Thus, this study cannot conclude the indications or appropriateness of interventions. With regards to specific surgical management data, the TriNetX database is limited in only being able to provide data used to form patient cohorts based on physician or hospital-inputted ICD-10 and CPT codes, and it cannot account for errors or variations in how physicians and medical systems may code or bill for certain procedures. Additionally, the study does not account for patient factors such as insurance status, socioeconomic status, comorbidities, or geographic location.

## Conclusions

TSFs are the most common long bone fractures in the United States and are associated with significant patient morbidity. Findings showing higher rates of ORIF compared to IMN were found in the study, which differs from other studies on the mainstay of treatment for TSF. This presents an opportunity for further studies to determine whether these findings reflect broader treatment trends, data sampling, or surgeon preference. Overall, TSFs treated with IMN were found to have lower rates of nonunion compared to ORIF. In addition, this study identified multiple findings regarding the demographics of patients who undergo either IMN or ORIF. IMN of TSF was more common in younger patients, while ORIF was more common in the older age groups. Males had higher rates of both IMN and ORIF when compared to female patients; however, both ORIF and IMN had similar rates of male-to-female utilization, indicating that both genders are being treated similarly with regard to the operative method of choice. Additionally, White patients and those who were not Hispanic or Latino had much higher rates of both IMN and ORIF compared to all other racial groups. Further efforts are needed to target these racial disparities in orthopedic care, as well as to look deeper at potential socioeconomic causes for these findings. 
